# Characteristics and genetic testing outcomes of patients with clinically suspected paraganglioma/pheochromocytoma (PGL/PCC) syndrome in Singapore

**DOI:** 10.1186/s13053-020-00156-9

**Published:** 2020-12-11

**Authors:** Kay Reen Ting, Pei Yi Ong, Samuel Ow Guan Wei, Rajeev Parameswaran, Chin Meng Khoo, Doddabele Srinivasa Deepak, Soo-Chin Lee

**Affiliations:** 1 Department of Haematology-Oncology, National University Cancer Institute, Singapore (NCIS), National University Health System, 1E Kent Ridge Road, Singapore, 119228 Singapore; 2 Yong Loo Lin School of Medicine, National University of Singapore, Singapore, Singapore; 3 Department of Surgery, Division of General Surgery (Thyroid and Endocrine Surgery), National University Hospital, Singapore, Singapore; 4 Department of Medicine, Division of Endocrinology, National University Hospital, Singapore, Singapore; 5 Cancer Science Institute, Singapore, Singapore

**Keywords:** Hereditary paraganglioma, Pheochromocytoma, Genetic testing, *SDHB*, *SDHD*, Chinese

## Abstract

**Background:**

Hereditary paraganglioma (PGL) and pheochromocytoma (PCC) syndromes are rare conditions, with limited data on spectrum of causative gene variants of these syndromes in Asian patients.

**Methods:**

We describe the clinical characteristics and genetic testing outcomes of patients with suspected hereditary PGL/PCC who were referred to a tertiary cancer genetics clinic in Singapore.

**Results:**

Among 2196 patients with suspected hereditary cancer syndrome evaluated at the cancer genetics clinic from 2000 to 2019, 13/2196 (0.6%) patients fulfilled clinical suspicion for hereditary PGL/PCC syndrome. After genetic counselling, 10 patients underwent multi-gene next generation sequencing and deletion/duplication analysis, including *SDHAF2, SDHA, SDHB, SDHC, SDHD, VHL, NF1, RET, MAX,* and *TMEM127*. Seven of 10 patients (70%) were identified to carry pathogenic variants, including 3 unrelated Chinese patients with head and neck PGL who carried the same *SDHD*: c.3G > C (p.Met1Ile) variant that was previously reported to be a possible founder variant in Chinese, and 3 patients with urogenital PGL and 1 patient with retroperitoneal PGL who carried different *SDHB* variants. Variant carriers were younger, more likely to present with multiple tumours, or have family history of paraganglioma or pheochromocytoma, than non- variant carriers.

**Conclusion:**

Hereditary PGL/PCC accounts for only 0.6% of patients seen in an adult cancer genetics clinic in Asia. *SDHD* and *SDHB* genes remain the most important causative genes of hereditary PGL/PCC in Asia even when patients are tested with multi-gene NGS panel.

## Introduction

Pheochromocytomas (PCC) and paragangliomas (PGL) are rare neuroendocrine tumours with incidence rate occurring around 2 to 8 cases per million person years [1, 2], affecting both genders equally and commonly occurring in the third to fifth decades of life. The 2017 World Health Organisation (WHO) classification of Endocrine Tumour and American Joint Committee on Cancer has classified these tumours based on the anatomy and biochemical features [3]. Head and neck paragangliomas (HN-PGL) involve the skull base, neck and upper mediastinum; with the commonest site of tumour occurring above the bifurcation of carotid arteries. This group of paragangliomas are typically parasympathetic and non-secretory. Extra-adrenal paragangliomas found in the lower mediastinum, abdomen such as the organ of Zuckerkandl and pelvis (thoraco-abdominal PGL) are typically catecholamine secretors resulting in clinical manifestation of sympathetic-like symptoms. Majority of PGL/PCC are benign cases but approximately 10% of PCC and around 15–35% of extra-adrenal abdominal paragangliomas are malignant [4, 5]. Malignant PGL/PCC may metastasize to bone, liver and lung, with predicted 5-year survival rates ranging from 12 to 80% [6, 7]; a meta-analysis reports the overall 5-year mortality rates for patients with metastatic PGL/PCC at 37% [8].

Historically, about 10% of PGL/PCCs were associated with hereditary syndromes, but recent discoveries have shown that at least 25–40% of PGL/PCC are linked to hereditary syndromes with several identified causative genes [9–11]. The classic hallmarks for hereditary PGL/PCC include an early age of onset, extra-adrenal disease, multiple primary tumours and metastatic tumours. Hereditary PGL/PCCs tend to present about 15 years younger than sporadic cases [12, 13].

There are at least 10 genetic syndromes with predisposition to PGL/PCC. The most common are the paraganglioma syndromes due to variants in the succinate dehydrogenase subunit (*SDH*) genes, *SDHD, SDHAF2, SDHC, SDHB* and *SDHA*, respectively, that are categorised into 5 types (PGL 1–5), with distinct clinical phenotypes [14]. Among these, variants in PGL1 (*SDHD*) and PGL4 (*SDHB*) are most commonly encountered in clinical practice. PGL1 (*SDHD*) is predominantly associated with head and neck PGL that frequently presents as multifocal disease and rarely malignant, while PGL4 (*SDHB*) presents with head and neck or thoraco-abdominal PGLs with only 20–25% being multifocal but behave more aggressively with ~ 30% being malignant. Adrenal pheochromocytomas and renal cell carcinomas occur in both PGL1 and PGL4, at ~ 10–25% and 8–14% respectively, while gastrointestinal stromal tumours have been reported in PGL1, 3, 4 and 5. In comparison, PGL2 presents almost exclusively with head and neck PGL with rarely other manifestations [14].

Other well-known hereditary syndromes associated with PGL/PCC include multiple endocrine neoplasm type 2 (MEN2), von Hippel-Lindau disease (VHL) and neurofibromatosis type 1 (NF1). In the last decade, newer genes such as *MAX* and *TMEM127* were reported to contribute to hereditary pheochromocytoma and paragangliomas. *MAX* variants are almost exclusively identified in patients with adrenal pheochromocytoma that are frequently bilateral [15], while *TMEM127* variant carriers most commonly present with single adrenal pheochromocytoma, and occasionally multiple head and neck or thoraco-abdominal PGLs.

Studies describing the causative variants and characteristics of hereditary PGL/PCC syndromes in Southeast Asia are limited. We describe a series of patients with suspected hereditary PGL/PCC syndrome who underwent multi-gene panel testing at a Cancer Genetics Program at an academic cancer centre in Singapore.

## Materials and methods

### Study group

A total of 2196 patients with suspected hereditary cancer syndromes were referred to the National University Cancer Institute, Singapore (NCIS) Adult Cancer Genetic Clinic for genetic counselling and consideration of genetic testing from year 2000 to December 2019. Thirteen of 2196 individuals (0.6%) were patients with suspected hereditary paraganglioma or pheochromocytoma (PGL/PCC) syndrome, presenting either with young onset PGL or PCC at or before age 40, multiple PGL and/or PCCs, and/or family history of PGL/PCC. Patients received genetic counselling and were offered genetic testing using a multi-gene panel test with full-gene sequencing and deletion/duplication analysis using next-generation sequencing (NGS) technology, including *SDHAF2, SDHA* (sequencing changes only), *SDHB, SDHC, SDHD, VHL, NF1, RET, MAX,* and *TMEM127*, as well as causative genes of common hereditary breast and colorectal cancer syndromes, including *BRCA1/2, TP53,* and the mismatch repair genes (*MLH1*, *MSH2*, *MSH6* and *PMS2*). Cascade testing was offered to first-degree relatives in patients tested positive for pathogenic germline variants.

## Results

**Fig. 1:**
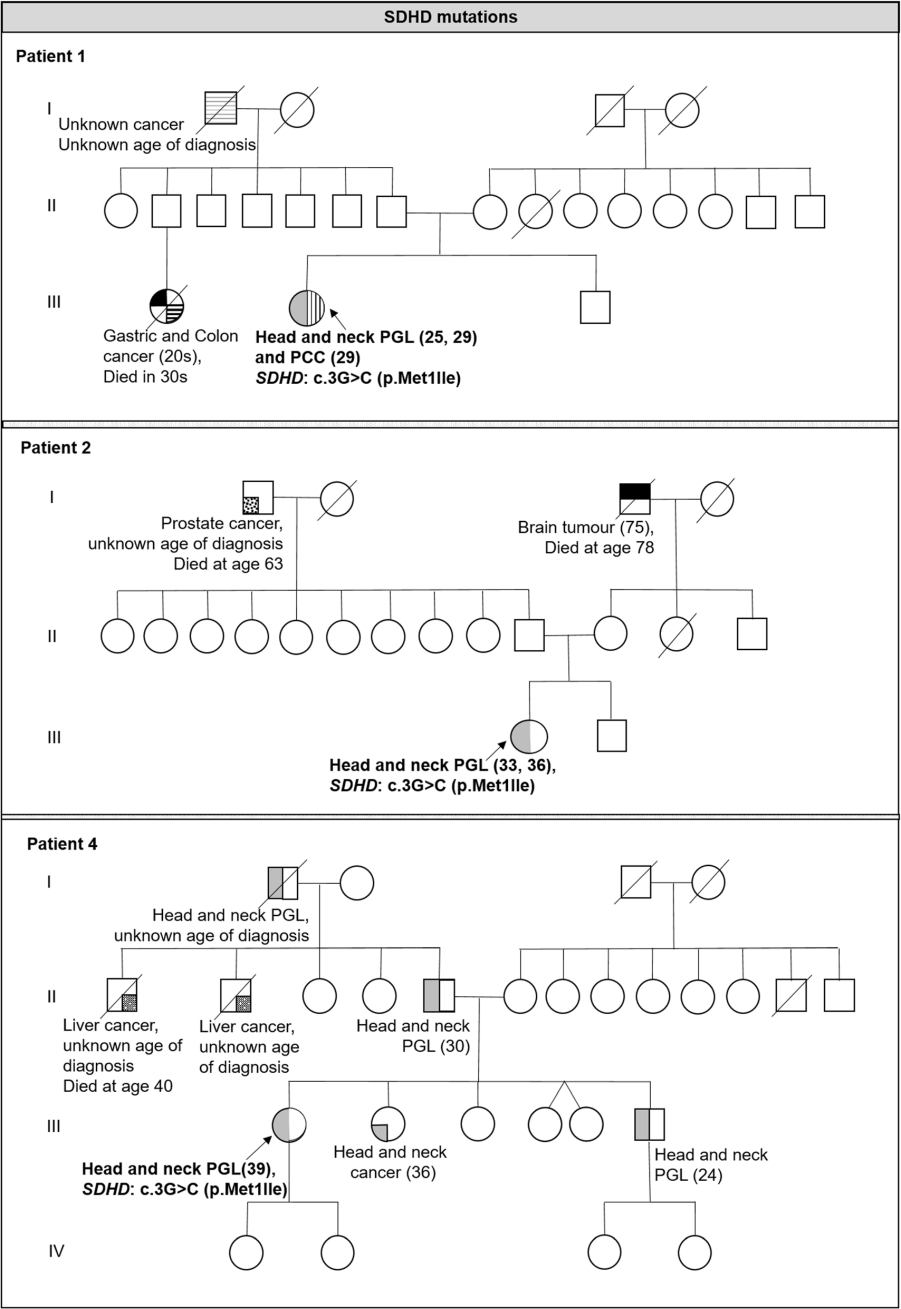
Family pedigree of 3 patients with positive *SDHD* variants

**Fig. 2:**
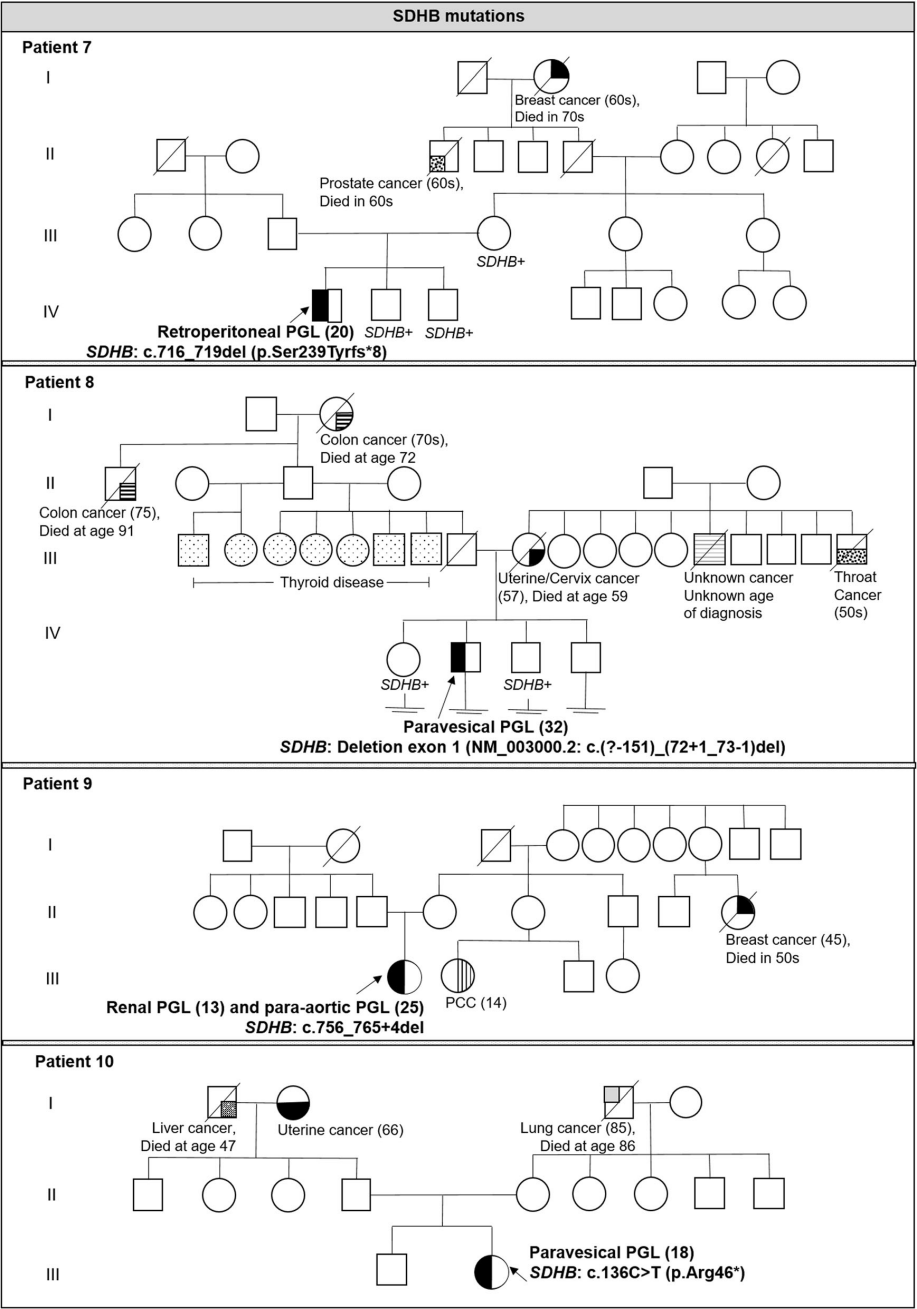
Family pedigree of 4 patients with positive *SDHB* variants

Table 1 summarizes the clinical characteristics of the 13 patients with suspected hereditary PGL/PCC syndrome and Figs. 1 and 2 shows the 7 family pedigree for those patients found with pathogenicvariants. Majority are female (*n* = 11, 84.6%) and Chinese (*n* = 9, 69.2%). Six patients (46.2%) presented with head and neck paraganglioma, five (38.5%) had extra-adrenal thoraco-abdominal paraganglioma with majority involving the urogenital tract (*n* = 4) and one involving the retroperitoneal region. The remaining 2 patients (15.4%) presented with adrenal pheochromocytoma alone. Median age at first presentation is 30 years (range 13–73). Ten patients (76.9%) had young onset PGL/PCC at or before age 40, 4/13 patients (30.8%) presented with multiple PGL/PCC tumours, and 3/13 patients (23.1%) had metastatic disease involving the bone, lung and liver. Two patients (18.2%) had family history of paraganglioma (*n* = 1) or pheochromocytoma (*n* = 1).

**Table 1:** Clinical Characteristics and Genetic Test Results of patients with suspected hereditary paraganglioma/pheochromocytoma syndrome (*n* = 13)

No	Patient profile	Age at diagnosis	Type of tumour	Tumour location	Multifocal lesions	Malignant (metastatic site)	Clinical features at presentation	Family history of pheochromocytoma or paraganglioma	Family history of other cancers	Gene	Genetic Variants
1	Chinese Singaporean female	25	Head and neck paraganglioma	Bilateral glomus jugulare	Yes	No	Incidental finding from radiological scan (Raised catecholamine levels)	Absent	Present Paternal first cousin: Gastric & Colon cancer - 20s, Paternal grandfather: unknown cancer and age of diagnosis	*SDHD*	NM_003002.3: c.3G > C (p.Met1Ile)
		29	Head and neck paraganglioma	Unilateral carotid body							
			Adrenal pheochromocytoma	Unilateral adrenal							
2	Chinese Indonesian female	33	Head and neck paraganglioma	Unilateral glomus jugulare	Yes	No	Hearing loss (Raised dopamine alone)	Absent	Present Maternal grandfather: Brain cancer - 75, Paternal grandfather: Prostate cancer - 63	*SDHD*	NM_003002.3: c.3G > C (p.Met1Ile)
		36	Head and neck paraganglioma	Bilateral carotid bodies and mediastinal masses							
3	Eurasian female	73	Head and neck paraganglioma	Multiple mediastinum masses (para-aortic), diaphragmatic crux masses and bilateral adrenals	Yes	No	Incidental finding from radiological scan (Normal catecholamine levels)	Absent	Present Sister: Breast cancer - 30, Mother: Colon cancer - 75, Maternal uncle: Lung cancer unknown age of diagnosis, Maternal first cousin: Breast cancer - 69		No pathogenic variants
4	Chinese Myanmese female	39	Head and neck paraganglioma	Unilateral carotid body	No	No	Hypertension (Normal catecholamines levels)	Present Brother: carotid PGL - 24, Father: neck PGL - 30, Paternal grandfather: possibly neck PGL, age unknown	Present Sister: head and neck cancer −36, Two paternal uncles: Hepatocellular cancer, age unknown	*SDHD*	NM_003002.3: c.3G > C (p.Met1Ile)
5	Chinese Singaporean female	30	Head and neck paraganglioma	Unilateral carotid body	No	No	Neck swelling (Normal catecholamines levels)	Absent	Absent		No pathogenic variants
6	Malay Singaporean female	24	Head and neck paraganglioma	Unilateral carotid body	No	Yes (Bone, lung)	Neck swelling (Normal catecholamines levels)	Absent	Present Mother: Breast cancer - 46, Maternal aunt: Breast cancer - 20-30s, Maternal uncle: probable Prostate cancer age 60s, Maternal first cousin: Breast cancer - 40s		Not tested
7	Filipino male	20	Non head and neck extra-adrenal paraganglioma	Retroperitoneal region	No	No	Hypertension (Raised noradrenaline and normetanephrine levels)	Absent but family members are *SDHB* variant carriers Mother: cancer free - 42, Brothers: cancer free – 13 and 8	Present Maternal great grandmother: Breast cancer – 60s Maternal granduncle: prostate cancer – 60s	*SHDB*	NM_003000.2: c.716_719del (p.Ser239Tyrfs*8)
8	Chinese male from Malaysia	32	Non head and neck extra-adrenal paraganglioma	Paravesical	No	No	Hypertension, palpitations (Raised noradrenaline and normetanephrine levels)	Absent but family members are *SDHB* variant carriers Sister: cancer free −34, Brother: cancer free – 28	Present Mother: Uterine/Cervical cancer - 57 Maternal uncle: Throat cancer - 50s Maternal uncle: Unknown cancer and age of diagnosis Paternal great grandmother: Colon cancer – 70s Paternal granduncle: Colon cancer - 75	*SDHB*	NM_003000.2: c.(?-151)_(72 + 1_73–1)del
9	Chinese- Myanmese female	13	Non head and neck extra-adrenal paraganglioma	Unilateral renal	Yes	Yes (Retroperitoneal and liver)	Hypertension, syncope (Raised noradrenaline and normetanephrine levels)	Present Maternal first cousin probable PCC - 14	Present Maternal aunt: Breast cancer - 45	*SDHB*	NM_003000.2: c.756_765 + 4del
		25	Non head and neck extra-adrenal paraganglioma	Para-aortic region							
10	Chinese Singaporean female	18	Non head and neck extra-adrenal paraganglioma	Paravesical	No	Yes (Lymph nodes and bone)	Hypertension, excessive sweating and headache (Raised noradrenaline and normetanephrine)	Absent	Present Maternal grandfather: Lung cancer – 85, Paternal grandfather: Liver cancer – unknown age of diagnosis, Paternal grandmother: Uterine cancer - 66	*SDHB*	NM_003000.2: c.136C > T (p.Arg46*)
11	Malay Singaporean female	63	Primary Central Nervous System lymphoma		No	No	Incidental finding from radiological scan (Normal catecholamine levels)	Absent	Absent		No pathogenic variants
		66	Non head and neck extra-adrenal paraganglioma	Bladder							
		65	Sigmoid adenocarcinoma, Stage III								
12	Chinese Singaporean female	29	Adrenal pheochromocytoma	Unilateral adrenal	No	No	Incidental finding from radiological scan (Raised catecholamine levels)	Unknown	Unknown		Not tested
13	Chinese Singaporean female	54	Adrenal pheochromocytoma	Unilateral adrenal	No	No	Incidental finding from radiological scan (Unknown catecholamine levels)	Absent	Present Maternal first cousin: Leukaemia - 12, Paternal first cousin: Head and neck cancer - 30		Not tested
			Clear cell renal cell carcinoma	Renal							

*PCC* Pheochromocytoma, *PGL* Paraganglioma

Patients 1–3 presented with multifocal HN-PGLs with patient 1 also presenting with adrenal pheochromocytoma and were suspected clinically to have PGL1. Three patients (Patients 4–6) presented with non-multifocal HN-PGL with no other manifestations and were suspected clinically to have PGL1 or PGL3. Five patients (Patients 7–11) presented with extra-adrenal thoraco-abdominal PGLs with two presenting with malignant disease. All five were clinically suspected to have PGL4. A differential diagnosis of Lynch syndrome was considered in Patient 11 in view of her personal history of multiple other malignancies including colorectal cancer. Patients 12 and 13 presented with adrenal pheochromocytoma with clinical features of hypersecretory catecholamines, with patient 13 presenting also with clear cell renal cell carcinoma, suspicious of PGL1, PGL4, or von Hippel-Lindau syndrome.

Ten of 13 patients (76.9%) underwent genetic testing. Seven patients (70.0%) were found to carry pathogenic variants (*SDHB* = 4, *SDHD* = 3). No other pathogenic variants were identified. Patients who tested positive for *SDHB* or *SDHD* pathogenic variants were younger than those who tested negative (mean age 26 ± 4 vs 55 ± 13 years, *p* = 0.015). All seven patients found with pathogenic variants were diagnosed with PGL/PCC clinically before age 40. Three out of four patients who had multifocal tumour (*n* = 3/4, 75.0%), two of three patients who presented with metastatic disease (*n* = 2/3, 66.7%), and both patients with family history of PGL/PCC (*n* = 2/2, 100.0%) and who underwent genetic testing, tested positive. Three of 13 patients (23.1%) did not undergo genetic testing as they were not keen to know the genetic information.

Among the seven patients found with pathogenic variants, 3 patients were found to carry pathogenic *SDHD* variants, including 2 Chinese patients and 1 Chinese-Myanmese patient. These three unrelated individuals all presented with head and neck paragangliomas, and were found to carry the same *SDHD*: c.3G > C (p.Met1Ile) variant that was previously reported to be a possible founder variant in Chinese [16–19]. Four patients were found to carry pathogenic *SDHB* variants, and all four presented with extra-adrenal thoraco-abdominal paragangliomas, including 3 of the 4 patients with urogenital tract involvement. These 4 unrelated patients had different *SDHB* variants, including three clearly pathogenic variants (large deletion = 1, nonsense variant = 1, frameshift variant leading to premature stop signal = 1) and a possibly pathogenic splice donor variant. All *SDHB* variants identified exist in ClinVar.

Two adult family members of Patient 7 and three adult family members of Patient 8 who were diagnosed with pathogenic *SDHB* variants underwent cascade testing; 3 were confirmed to carry the familial *SDHB* pathogenic variants (mean age 35 years, range 28–42). All 3 family members were asymptomatic and cancer free, including, of note, the 42-year old mother of Patient 7.

## Discussion

Hereditary paraganglioma or pheochromocytoma syndromes are rare conditions, with fewer than 1% of patients who were referred to our adult cancer genetics clinic fulfilling clinical suspicion for hereditary PGL/PCC. While *SDHB, SDHC* and *SDHD* genes are the classical causative genes of hereditary PGL/PCC, advancements in genetic analysis have revealed rarer causative genes such as *SDHA, SDHAF2, MAX* and *TMEM127*. The Task Force committee consisting of members from The Endocrine Society, European Society of Endocrinology, and American Association for Clinical Chemistry have reviewed the diagnostic algorithms from various studies and has recommended an optimal strategy for genetic screening, generally prioritizing younger age individuals, those with positive family history, and/or multifocal PGL/PCC, to undergo genetic tests [20]. Individuals with clinically high-risk features who do not show variant in the classic genes should be tested for the rarer genes [15], while targeted germline variant testing of *RET*, neurofibromatosis type 1 (*NF1*) or von Hippel-Lindau syndrome (*VHL*) are considered for individuals with syndromic presentation.

Although hereditary PGL/PCC is a fairly distinct entity, several of our patients report family history of other carcinomas that are unrelated to PGL/PCC, including breast, colorectal, prostate, and leukaemia, suggesting differential diagnoses such as *BRCA1/2* hereditary breast cancer syndrome, hereditary colorectal cancer syndrome, and Li Fraumeni syndrome. This highlights the benefits of next-generation sequencing multigene panel testing in these patients that encompasses not only genes associated with hereditary PGL/PCC syndrome but also causative genes of other adult hereditary cancer syndromes. In our highly selected population, 70% of patients tested were found to carry pathogenic variants confirming the diagnosis of hereditary PGL/PCC. Interestingly, despite the broad-based testing approach, pathogenic variants were only identified in the two most important *SDH* genes, namely *SDHD* and *SDHB*, with no pathogenic variants identified in any other PGL/PCC causative genes or other hereditary cancer genes. This observation is largely attributed to the small sample size, but also ascertains the importance of the *SDHD* and *SDHB* genes as causative genes of hereditary PGL/PCC in Asia. This has been similarly reported in another study conducted in Singapore, in which 5 of 7 patients with suspected hereditary PGL/PCC who tested positive carried *SDHD* (*n* = 2) or *SDHB* (*n* = 3) pathogenic variants, with the remaining two patients harbouring *VHL* pathogenic variants [21].

Among the seven patients who were found with pathogenic germline variants in our series, 3 unrelated patients carried the same *SDHD*: c.3G > C (p.Met1Ile) variant, while 4 patients were found with different *SDHB* variants. *SDHD* and *SDHB* variant carriers have distinct characteristics, with *SDHD* carriers typically presenting with head and neck PGLs that are rarely malignant, and *SDHB* carriers having lesions involving mostly extra-adrenal non-head and neck sites that behave more aggressively. Consistent with many other reports [21–23], patients who have younger onset presentation, multiple tumours, metastatic disease or family history of PGL/PCCs, were more likely to be diagnosed with pathogenic *SDH* variants in our study. Interestingly, four-fifths of patients in our series found with pathogenic *SDHB* variant presented with urogenital PGL (renal = 1, bladder = 3); three were Chinese and the other was of mixed Chinese-Myanmese heritage. While genitourinary paragangliomas have been described, it is uncommon and comprises only 6.7% of all PGL cases in the US population [24]. Among urogenital PGLs, bladder (83.3%) is the commonest site followed by other sites like renal, renal pelvis or spermatic cord. In the Surveillance, Epidemiology, and End Results (SEER) database, genitourinary PGLs are frequently seen in male and younger individuals, consistent with what we observed in our study [24]. A study conducted in London on patients with bladder paraganglioma found pathogenic *SDHB* variants in 6/9 individuals, again mostly in male and younger individuals [25]. Although there have been several case series on urogenital paragangliomas in Asia, there is limited information on causative germline variants of these rare tumours [26, 27]. In the few Asian genetic studies on urogenital paragangliomas, *SDHB* exon 7 deletion was reported in an Indian patient with bladder paraganglioma [28], while *SDHB*: c.112delC (p.Arg38fs) variant was reported in a Hong Kong Chinese patient with recurrent metastatic bladder paraganglioma [29]. Contrary to our study in which three of four patients who tested positive for pathogenic *SDHB* variants had urogenital PGL, a Korean study of 2 patients with pathogenic *SDHB* variants did not report any urogenital paraganglioma involvement [30]; instead, both patients presented with adrenal pheochromocytoma with one of them behaving in a malignant manner. More studies are required in Asia to determine if urogenital paragangliomas due to *SDHB* variants are more common in certain ethnic populations.

In our series, only 15.4% (2/13) patients reported family history of PCC and/or PGL. Yet, 70% (7/10) patients who underwent genetic testing were confirmed to carry pathogenic *SDHD* or *SDHB* variants. Both patients with family history of PGL/PCC tested positive, underscoring the importance of family history as a predictor for pathogenic SDH variants. The low proportion of reported family history may be due to small family sizes, ascertainment bias, and maternal genomic imprinting reported with some *SDH* genes, and highlights the importance of not relying only on family history to select individuals for hereditary PGL/PCC testing in the clinic.

Separately, our series had identified several asymptomatic first-degree relatives to be *SDHB* variant carriers from two families (Patients 7 and 8). Of note, Patient 7 was a 20-year old Filipino male who presented with retroperitoneal paraganglioma associated with hypertension from raised noradrenaline and normetanephrines but reports no family history of PGL or PCC. Upon confirmation of a pathogenic *SDHB* variant in him, his cancer-free mother, aged 42, underwent cascade testing and was confirmed to carry the same variant, providing evidence of the maternal origin of the variant. Patient 8 is a 32-year old Chinese male who presented with paravesical paraganglioma associated with hypertension from raised nonadrenaline and normetapherines and again reported no definitive family history of PGL/PCC. Although his parents were not directly tested, two of his adult siblings subsequently tested positive for the same variant, confirming that the variant must have been inherited rather than occurring de novo. Recent studies have reported a lower range of penetrance of 25–50% in *SDHB* variant carriers after taking into account the ascertainment of variant carriers [31, 32]. The lower penetrance may be another reason for the lack of family history in the majority of patients seen in our series.

*SDH* variants are generally distributed along the entire genes, with no obvious hot spots. However, a small number of founder variants in the *SDH* genes that occur in high frequencies in certain geographically or culturally isolated groups of people have been reported, mostly amongst the European population. For example, *SDHD* founder variants were reported amongst the Dutch (*SDHD*: c.274G > T (p.Asp92Tyr)) [33, 34], Polish (*SDHD*: c.33C > A (p.Cys11*)) [35], and Italian populations (*SDHD*: c.325C > T (p.Gln109*)) [36], while an *SDHB* founder variant (*SDHB*: c.201-4429_287-933del (p.Cys68Hisfs*21)) has been reported in the Dutch population [37]. These founder variants have generated considerable interest, because they might direct testing strategy towards specific prevalent founder variants in certain population. In comparison, there have been limited reports of SDH founder variants in Asians. Zha et al reported the *SDHD*: c.3G > C (p.Met1Ile) variant as a possible founder variant in the Chinese population [17], with haplotype analysis showing three out of four unrelated Chinese families carrying the *SDHD*: c.3G > C (p.Met1Ile) variant residing in China, Singapore [16] and Hong Kong [18], to share a common haplotype spanning a 280 kb region. In our current study, all three patients who were diagnosed with *SDHD* pathogenic variants carried the same *SDHD*: c.3G > C (p.Met1Ile) variant, which results in abolishment of the initiation codon. These three unrelated patients were of Chinese descent, although they live in different parts of South-East Asia: Singapore, Indonesia and Myammar, further supporting the previous observation that this variant is a common founder variant in Chinese. Interestingly, we were not initially aware of the Chinese ancestry of the Myanmese patient whose variant is likely paternal in origin as both her paternal grandfather and father presented with head and neck paragangliomas. After she was diagnosed to carry the *SDHD*: c.3G > C (p.Met1Ile) variant, further questioning revealed that she was of mixed Chinese-Myanmese ancestry, with her affected paternal grandfather being Chinese. Maternal imprinting has been reported for *SDHD *variants, and the clinical manifestation of PGL in this index patient whose variant is paternal in origin is consistent with this notion. The patient as well as her affected brother each has two teenage children who can eventually undergo predictive testing; it will be of interest to follow up this family closely to determine the transmission pattern of the *SDHD*: c.3G > C (p.Met1Ile) variant.

## Conclusion

Hereditary PGL/PCC is rare, accounting for around 0.6% of patients encountered in an adult cancer genetics clinic in Asia. Fewer than 20% of patients report family history of PGL/PCCs, highlighting the importance of other clinical features such as young age at diagnosis, multiple tumours and metastatic disease to identify high-risk individuals for genetic testing. The increasing availability of multi-gene panel testing with next generation sequencing has facilitated the diagnosis of pathogenic variants in these individuals. *SDHD* and *SDHB* genes remain the most important causative genes of hereditary PGL/PCC in Asia, with an *SDHD* founder variant existing in Chinese head and neck PGL families who reside in different countries in Asia.

## Data Availability

All data generated or analyzed during this study are included in this published article and its supplementary information files.
